# Presaturation Power Adjusted Pulsed CEST: A Method to Increase Independence of Target CEST Signals

**DOI:** 10.1155/2018/3141789

**Published:** 2018-05-08

**Authors:** Kazufumi Kikuchi, Keisuke Ishimatsu, Shanrong Zhang, Ivan E. Dimitrov, Hiroshi Honda, A. Dean Sherry, Masaya Takahashi

**Affiliations:** ^1^Advanced Imaging Research Center, University of Texas Southwestern Medical Center, Dallas, TX, USA; ^2^Philips Healthcare, Gainesville, FL, USA; ^3^Department of Clinical Radiology, Graduate School of Medical Sciences, Kyushu University, Fukuoka, Japan

## Abstract

Chemical exchange saturation transfer (CEST) imaging has been demonstrated to discuss the concentration changes of amide proton, glutamate, creatine, or glucose measured at 3.5, 3.0, 2.0, and 1.0–1.2 ppm. However, these peaks in *z*-spectra are quite broad and overlap with each other, and thus, the independence of a CEST signal on any specific metabolite is still open to question. Here, we described whether there was interference among the CEST signals and how these CEST signals behave when the power of the presaturation pulse was changed. Based on these results, further experiments were designed to investigate a method to increase the independence of the CEST signal in both phantoms and animals. The result illustrates a clear interference among CEST signals. A presaturation power adjusted pulsed- (PPAP-) CEST method which was designed based on the exchange rates of the metabolites can be used to remove contributions from other exchanging species in the same sample. Further, the method was shown to improve the independence of the glutamate signal *in vivo* in the renal medulla in mice. The PPAP-CEST method has the potential to increase the independence of any target CEST signals *in vivo* by choosing the appropriate combination of pulse amplitudes and durations.

## 1. Introduction

Chemical exchange saturation transfer (CEST) imaging has drawn considerable attention as a novel mechanism of MRI contrast. Unlike conventional MRI methods, this method provides unique information about the presence of certain metabolites or classes of molecules that exchange protons with bulk water protons in tissues [1, 2]. Amide proton transfer (APT) imaging, a subset of CEST imaging, refers specifically to exchange of –NH protons on endogenous mobile proteins and peptides. APT imaging is becoming widely used because it provides new insights into the molecular status of tumors [3, 4]. Many groups have shown that APT imaging provides more sensitive detection of tumors compared to conventional MRI methods in both animal models [5–7] and human cases [4, 8]. Furthermore, the method detects early responses to therapy well before any anatomical changes are apparent [7]. Recently, CEST imaging has been extended to investigate other metabolites, and more studies have discussed the changes in tissue concentration of glutamate [9], creatine [10], or glucose [11] in several diseases [12, 13].

To obtain a CEST image, a *z*-spectrum is first collected by applying a train of presaturation pulses across a range of resonance frequencies, typically ±5–7 ppm with water set to 0 ppm, to identify all endogenous exchanging protons in a tissue of interest. Subsequently, the CEST signal of any metabolites containing an exchanging proton is detected by subtracting the water intensity at each ±*X* frequency to identify each molecular class of protons in exchange with water [14]. For example, proteins/peptides [3], glutamate [9], creatine [10], and glucose [11] contain exchanging amide (–NH), amine (–NH_2_), guanidine [HN=C(NH_2_)_2_], and hydroxyl (–OH) protons, respectively. Thus, the CEST spectrum of those molecular classes would show CEST exchange peaks at 3.5, 3.0, 2.0, or 1.0–1.2 ppm, respectively, again with water protons set to 0 ppm. Such CEST exchange peaks are commonly detected in a *z*-spectrum by performing an asymmetry analysis and presented as a magnetization transfer asymmetry ratio (MTR_asym_) or by fitting analysis using some mathematical functions like Gaussian and/or Lorentzian curve fitting methods. Even though specific CEST exchange peaks can be identified in either an asymmetry analysis or a fitting analysis, these peaks are often quite broad and overlap with each other. Therefore, the independence of a CEST signal on any specific metabolite is still open to question [15]. Nevertheless, most CEST studies focus on signal changes at a given frequency and often do not consider the impact of neighboring overlapping signals.

The purpose of this study was to investigate whether the CEST signal from a given metabolite is affected by the neighboring CEST signals of other common metabolites identified from 0 to 3.5 ppm in tissue. For this purpose, we first evaluated in phantoms (1) whether there was interference among the CEST signals of protein/peptide, glutamate, creatine, and glucose at a common set of CEST imaging parameters and (2) how these CEST signals behave when the power (amplitude and duration) of the presaturation pulse was changed. Based on these results, further experiments were designed to investigate a method to increase the independence of the CEST signal in both phantoms and animals. The latter study focused on glutamate, an important metabolite *in vivo*, and its detection by CEST in the mouse kidney during renal filtration.

## 2. Materials and Methods

### 2.1. MR System

All studies were conducted in a 7.0 T experimental system with a 35 mm inner diameter Horizontal Millipede coil (Agilent, Palo Alto, CA).

### 2.2. Phantom Study

**Figure 1:**
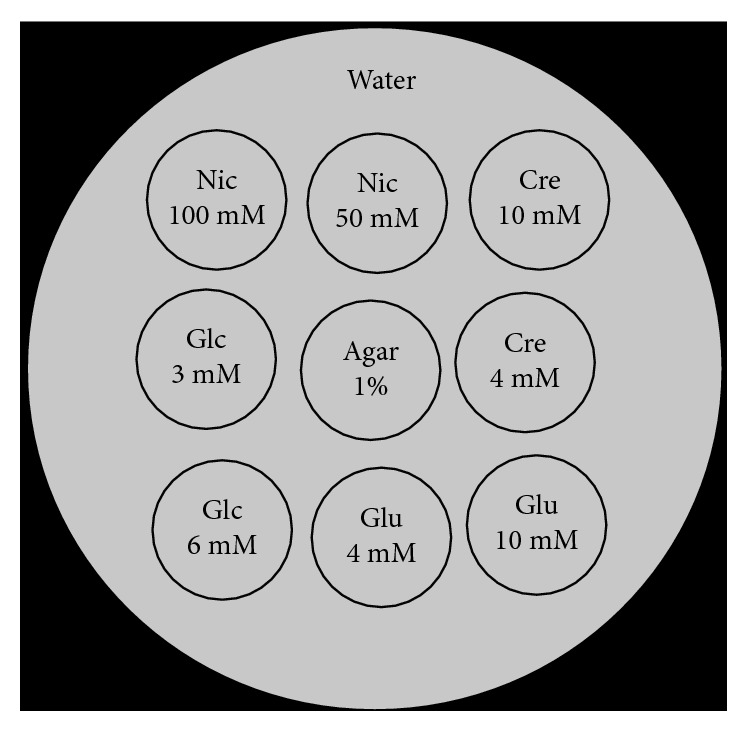
The phantoms including nicotinamide (Nic), glutamate (Glu), creatine (Cre), and glucose (Glc) aqueous solution in low and high concentrations. A 1% agar phantom is also included as a control. The low and high concentrations for each substance are determined to show approximately 5% and 10% CEST signals at 3.5, 3.0, 2.0, and 1.0 ppm, respectively, in a condition of CEST imaging at 37°C.

We prepared eight separate phantoms containing nicotinamide (Nic), glutamate (Glu), creatine (Cre), and glucose (Glc) aqueous solution in low and high concentrations with distilled water. The low versus high concentrations for each substance were chosen to yield approximately 5% and 10% CEST signals at 3.5, 3.0, 2.0, and 1.0 ppm, respectively, using the imaging parameters previously used for CEST imaging in mice [7] at 37°C. The concentrations established to meet these conditions were as follows: Nic, 50 and 100 mM; Glu, 4 and 10 mM; Cre, 4 and 10 mM; and Glc, 3 and 6 mM (Figure 1). The pH of these phantoms was adjusted to 7.2. Several previous CEST phantom studies in the phantoms of Glu [9, 16], Cre [17, 18], and Glc [11, 19] were conducted at pH values ranging from 7.0 to 7.4. Thus, we selected a pH of 7.2 as a median value.

In the first phantom study, we evaluated whether there was interference among the CEST signals of protein/peptide, glutamate, creatine, and glucose at a common set of CEST imaging parameters in phantoms. For this purpose, the set of phantoms in a water-filled tube was positioned parallel to the magnetic field in a horizontal bore magnet to reduce any susceptibility effects. An axial gradient echo image (2 mm thickness with centric k-space ordering) was collected following a presaturation pulse (continuous wave: CW block pulse) with an amplitude of 2.3 *µ*T, and a duration of 5 s (2.3 *µ*T/5 s) was repeated at 61 different frequency offsets from 6 to −6 ppm with an interval of 0.2 ppm. Other imaging parameters were repetition time (TR)/echo time (TE) = 5.32/2.64 ms, flip angle = 20°, field of view (FOV) = 32 × 32 mm, matrix = 128 × 64 (reconstructed to 128 × 128), and number of excitations (NEX) = 4. A reference image was obtained with the presaturation pulse set to 300 ppm. Water saturation shift referencing (WASSR) images were collected with a CW pulse (0.2 *µ*T/200 ms) which was applied at 31 frequency offsets every ∼0.03 ppm. This WASSR imaging repeated both from 0.5 to −0.5 ppm and from −0.5 to 0.5 ppm, of which the results were averaged for *B*_0_ inhomogeneity corrections; otherwise, the saturation effect due to the short TR resulted in errors.

In the second phantom study, we evaluated how these CEST signals behave when the power (amplitude and duration) of the presaturation pulse was changed. To investigate the presaturation power-dependent changes in each CEST signal, CEST imaging was repeated using the same phantoms using different RF pulse amplitudes (1.28, 2.3, 3.83, 4.6, and 5.5 *µ*T) and durations (1, 3, and 5 s).

### 2.3. Animal Study

The protocols in the animal study were approved by the Institutional Animal Care and Use Committee. The study attempted a method to increase the independence of the Glu signal by diminishing an interference from a neighboring Nic signal in the kidney. We used 12 healthy male C57BL/6 mice (4-week-old, Charles River Laboratories, Inc., Wilmington, MA) under anesthesia with 1.5–2% of isoflurane (AERRANE, Baxter Healthcare Corporation, IL) mixed in 100% oxygen. Each animal was placed supine with a respiratory sensor in the center of the RF coil. The dose of isoflurane was adjusted occasionally to keep the respiratory rate constant, 20–22 per minute, and it was continuously monitored by an MRI-compatible small-animal respiratory gating device (SA Instruments, Stony Brook, NY).

We implemented a pilot study first to determine an appropriate Glu dose for the study to separate the Glu signal from the coinjected Nic signal in the CEST imaging. Each animal was subjected to the CEST imaging (5.5 *µ*T/3 s) before and after intraperitoneal (ip) injection of the Glu alone aqueous solution at a different dose (0.7, 1.4, 2.8, and 5.6 mmol/kg, *n*=1 per dose), respectively. Based on the result in the pilot study, two different mixed solutions were prepared for injection: both contained the same dose (1.4 mmol/kg) of Glu plus either a lower (0.7 mmol/kg; low-Nic, *n*=4) or higher dose (5.6 mmol/kg; high-Nic, *n*=4) of Nic. Both low-Nic and high-Nic solutions were adjusted to the pH of 7.4 and mixed with phosphate-buffered saline (PBS). Considering solubility in PBS, we used L-glutamic acid monosodium salt hydrate (Sigma-Aldrich Chemical Co, St. Louis, MO) and nicotinamide (Sigma-Aldrich Chemical Co, St. Louis, MO) for the injection solutions. The injection doses for Glu and Nic used in the present study were determined as tolerable doses (Glu: ∼5.9 mmol/kg; Nic: ∼16 mmol/kg) for small rodents demonstrated in the previous literature [9, 20–22].

### 2.4. Presaturation Power Adjusted Pulsed- (PPAP-) CEST Method

After scout localizer images were collected, coronal multislice T2-weighted images were obtained to identify the orientations of both kidneys using a fast spin-echo sequence: TR/TE = 2500/60 ms, FOV = 32 × 32 mm, matrix = 128 × 128, slice thickness = 1 mm, no intersection gap, fat suppression, and NEX = 4. For the CEST imaging, we selected a single 1.0 mm coronal imaging slab that depicted the center of either the right or left kidney. We defined the GluCEST map as the image of MTR_asym_ at 3.0 ppm [9]. Presaturation power adjusted pulsed- (PPAP-) CEST method aims at increasing independence of a target metabolite by using two or more presaturation pulses that have different amplitudes and/or durations. In the present study, a respiratory-gated CEST imaging was implemented using a PPAP-CEST method that used two CW block pulses with different amplitudes of 2.3 *µ*T and 5.5 *µ*T where a centric-ordered gradient-echo image was obtained following each presaturation pulse alternatively in each frequency offset from −5.0 to 5.0 ppm with an interval of 0.5 ppm (a total of 21 offsets). A reference image with the saturation offset at 300 ppm was also acquired, affording a total scan time of approximately 10 min. The duration of the presaturation pulses was adjusted to 2.5 s so that the image acquisition followed by a presaturation pulse was executed within the end-expiratory phase in each respiratory cycle. Each CEST imaging was repeated three times to evaluate the stability of CEST signals before injection (baseline) followed by a WASSR imaging. Subsequently, the same imaging was performed three times immediately up to 30 min after ip injection of Glu plus either low-Nic or high-Nic solution.

### 2.5. Image Analysis

The *z*-spectrum was fitted on a pixel-by-pixel basis according to the procedure on positive and negative sides of frequency offsets (phantom: 61 offsets and animal: 21 offsets) followed by a pixel-wise *B*_0_ inhomogeneity through interpolation and centering of the *z*-spectrum [23], respectively, by WASSR as previously reported [7]. The CEST signal is defined as the reduction in bulk water signal intensity that results from chemical exchange of water protons when a saturation pulse is applied at given frequencies; 3.5, 3.0, 2.0, and 1.0 ppm, respectively, with water set to 0 ppm. MTR_asym_ is defined as [SI (−*ω* ppm) – SI (+*ω* ppm)]/SI (offset, ∼300 ppm), where SI is the signal intensity on the images with presaturation pulse at each offset and *ω* is for the specific frequency of the metabolite in a *z*-spectrum to be referenced to water. CEST map was generated as the image of MTR_asym_ at a given specific frequency. In the animal study, the *z*-spectrum and MTR_asym_ were generated using the same method as described for the phantom study.

In the first phantom study, the four *z*-spectra from each substance at their respective “high” concentrations were summed to mimic a condition where all four substances would have been mixed together in one sample. This is referred to as the 4-high *z*-spectrum. To mimic a condition where the concentration of any one of the four substances is reduced, we also generated *z*-spectra that represented a sum of three *z*-spectra of three metabolites in high concentration and one *z*-spectrum of the remaining one metabolite in low concentration. On these five-summed *z*-spectra (4-high, Nic-low, Glu-low, Cre-low, and Glc-low), we measured the CEST signals at 3.5, 3.0, 2.0, and 1.0 ppm both in an asymmetry analysis and a Lorentzian fitting analysis. In Lorentzian fitting, one line shape was fitted to represent the metabolite signal, and the other line shape was fitted to represent the direct saturation of the water signal. We additionally measured the CEST signals in a phantom including all four substances at the high concentration (real 4-high), respectively, to compare with those in the virtual 4-high. In the second phantom study, we measured the CEST signals of 15 data sets (5 amplitudes × 3 durations) at 3.5, 3.0, 2.0, and 1.0 ppm for Nic, Glu, Cre, and Glc signals, respectively. In these phantom studies, the regions of interest (ROIs) were carefully placed on each phantom within a circular region of interest (ROI, ∼8 mm^2^, and 128 pixels).

In the animal study, we measured the CEST signals of the renal medulla at each time (0, 10, 20, and 30 min) in both Glu plus low-Nic and high-Nic injection groups, and all data were measured at 3.0 ppm as the GluCEST signal. The five ROIs were placed at every 45° from the superior (0°) to the posterior (180°) poles in the renal medulla within a circular region of interest (ROI, ∼2.5 mm^2^, 40 pixels). The average value for three ROIs after the maximal and minimal values of five measurements was excluded and was representative of the Glu signal in the kidney [24]. The measured values from three pre-imaging at 0 min were averaged to represent the “baseline” to compare with the CEST signals after injection.

All image processing was executed using MATLAB (R2016b, Math Works, Natick, MA) and ImageJ/Fiji (version 2.0.0-rc-59/1.51k, National Institutes of Health, Bethesda, MD).

### 2.6. Statistical Analysis

All values are expressed as mean ± standard deviation (SD). The temporal changes in the CEST signals at 3.0 ppm among three time points (10, 20, 30 min) were compared with the averaged baseline (three data at 0 min) using Dunnett's multiple comparison test. The CEST signals at 3.0 ppm were compared between the low-Nic and high-Nic using Student's *t*-test at each time point following the Shapiro–Wilk test in which the null hypothesis is that the data are normally distributed.

All statistical analyses were performed by using a commercially available software (Prism 7.0; GraphPad Software, La Jolla, CA), and a *P* < 0.05 was considered statistically significant.

## 3. Results

### 3.1. Phantom Study

**Figure 2:**
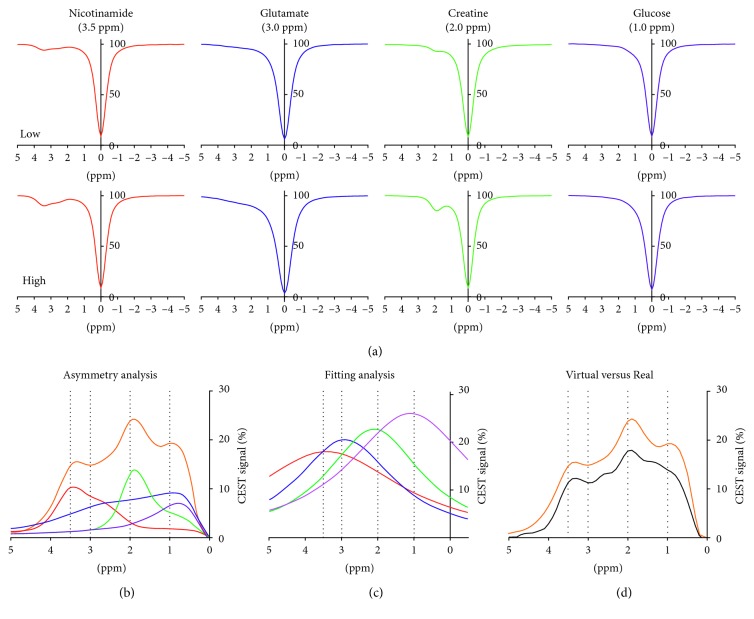
(a) The *z*-spectra exhibit a reduction in bulk water signal as a result of chemical exchange of water protons with each substance (nicotinamide at 3.5 ppm, glutamate at 3.0 ppm, creatine at 2.0 ppm, and glucose at 1.0 ppm, resp.). The results are showed in the magnetization transfer asymmetry ratio (MTR_asym_) analysis (b) and the fitting (Lorentzian) analysis (c). The orange line mimic the summed up MTR_asym_ curves of all four substances (virtual 4-high) (b). The MTR_asym_ in the virtual 4-high was similar to that in the real 4-high (virtual (orange) versus real (black)) (d).

**Figure 3:**
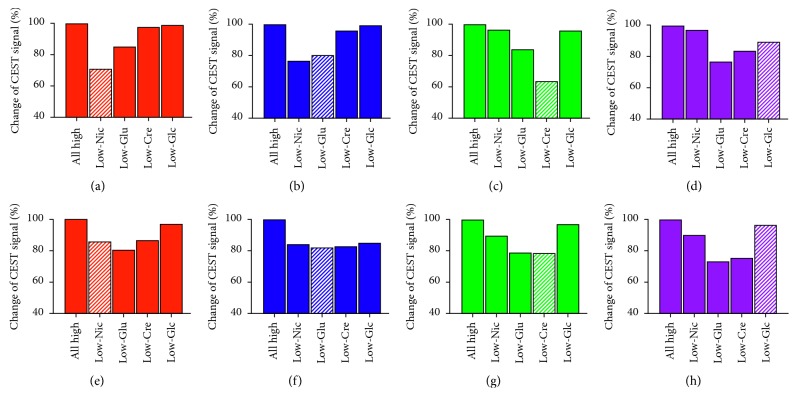
Percent change in the total CEST signal at each frequency when the concentration is reduced in any one substance relative to the signal in the 4-high *z*-spectra. Each signal is lowered when its own concentration is reduced (meshed-column) and when the concentration of most other substances is reduced. Compared to asymmetry analysis (a–d), the effect of CEST interference from neighboring signals was magnified in the fitting analysis (e–h). The results were obtained using a 2.3 *µ*T/5 s presaturation pulse at 37°C. (a) Nicotinamide at 3.5 ppm. (b) Glutamate at 3.0 ppm. (c) Creatine at 2.0 ppm. (d) Glucose at 1.0 ppm. (e) Nicotinamide at 3.5 ppm. (f) Glutamate at 3.0 ppm. (g) Creatine at 2.0 ppm. (h) Glucose at 1.0 ppm.

Figure 2 shows the four *z*-spectra of each substance at both low and high concentrations (Figure 2(a)). In the “4-high” *z*-spectrum, the intensity of the CEST signal at 3.5, 3.0, 2.0, and 1.0 ppm measured directly by asymmetry analysis (Figure 2(b), 4-high, orange) was 14.6% (Nic), 14.8% (Glu), 23.8% (Cre), and 19.5% (Glc), respectively. Each value was higher than those actually measured in the MTR_asym_ spectrum of individual substance (Figure 2(b); Nic, red; Glu, blue; Cre, green; Glc, purple) due to overlap of the MTR_asym_ spectrum of other substances. The MTR_asym_ in the virtual 4-high was similar to that in the real 4-high (Figure 2(d), virtual: orange versus real: black). When the 4-high MTR_asym_ spectra are compared with the MTR_asym_ spectra that mimic a condition and when concentration of any one of the four substances is reduced (Nic-low, Glu-low, Cre-low, and Glc-low), the CEST signal from each substance decreased not only when its own concentration was reduced but also when the concentration of any one of the other substances was reduced (Figure 3). For example, the Glu signal (Figure 3(b)) was lowered by 20% when the Glu concentration was reduced, but it was also lower by 24%, 4%, and 0.5% when the concentrations of any one of Nic, Cre, and Glc were reduced, respectively. This illustrates a clear interference of each CEST signal on the presence of any other substances in the same sample. The impact of CEST interference was even greater when the analysis was done by fitting the individual spectra to a Lorentzian line shape (Figures 3(e)–3(h)). This result was certainly not unexpected because the CEST peaks of each individual substance are well described by such simple nuclear magnetic resonance line shapes, so they are considerably broader than the individual MTR_asym_ spectra (cf. spectra in Figure 2(b) and 2(c)).

**Figure 4:**
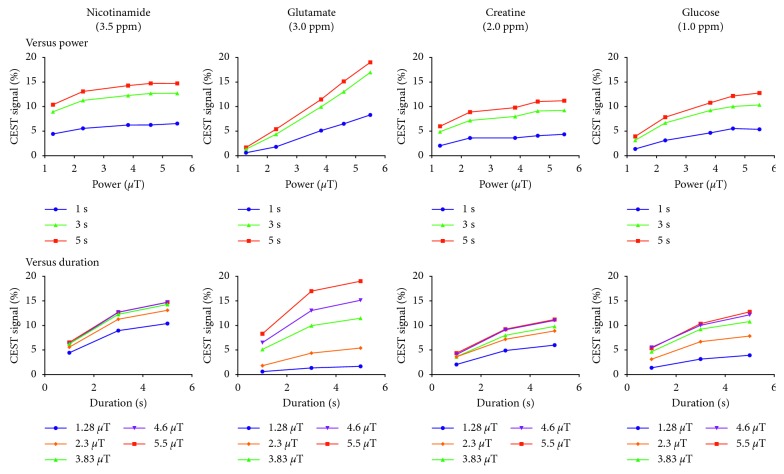
The changes of the CEST signal are shown when the amplitude and duration of presaturation pulses were changed. All signals increase as either the amplitude or duration of the presaturation pulse is increased as expected, and the amplitude and duration pulse dependencies differ for each substance. The average increments of the signal per 1 *µ*T through 2.3 *µ*T to 5.5 *µ*T for the substances are 0.4% in nicotinamide, 3.4% in glutamate, 0.5% in creatine, and 1.1% in glucose, respectively. Since the % change is somewhat higher for glutamate and glucose, these two substances are referred to as “amplitude-dependent.”

Figure 4 demonstrates the changes of the CEST signal when the amplitude and duration of presaturation pulses were changed. All signals increased as either the amplitude or duration of the saturation pulse was increased as expected, and the amplitude and duration dependencies differed for each substance. The average increments of the signal per 1 *µ*T through 2.3 *µ*T to 5.5 *µ*T for the substances were 0.4% in Nic, 3.4% in Glu, 0.5% in Cre, and 1.1% in Glc, respectively. Since the % change was somewhat higher for Glu and Glc (Figure 4), these two substances were hereafter referred to as “amplitude-dependent.”

**Figure 5:**
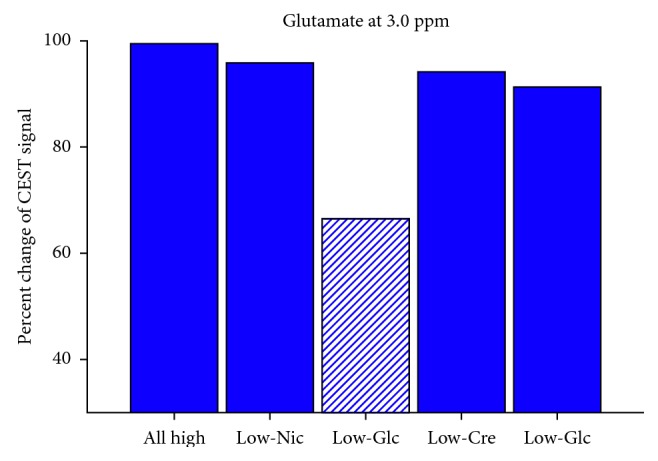
% change of the glutamate signal when the concentration is lowered in any one substance relative to the signal in the 4-high *z*-spectrum (asymmetry analysis). The signal is measured by the subtraction of 2.3 *µ*T/3 s from 5.5 *µ*T/3 s. The glutamate signal is reduced when its concentration is lowered (meshed column) but do not change much when the concentration of another substance is reduced.

Based on the results, we attempted to reduce the interference among CEST signals by subtraction of the images with different parameters. When the result at 2.3 *µ*T/3 s was subtracted from the result at 5.5 *µ*T/3 s, one finds that the Glu signal is much less affected by the CEST signals of the other substances (Figure 5). Compared to Figure 3(b), the Glu signal was lower only when the Glu concentration was reduced in this subtraction, and the changes in the Glu signal were relatively small when the concentrations of Nic, Cre, or Glc were reduced (Figure 5).

### 3.2. Animal Study

**Figure 6:**
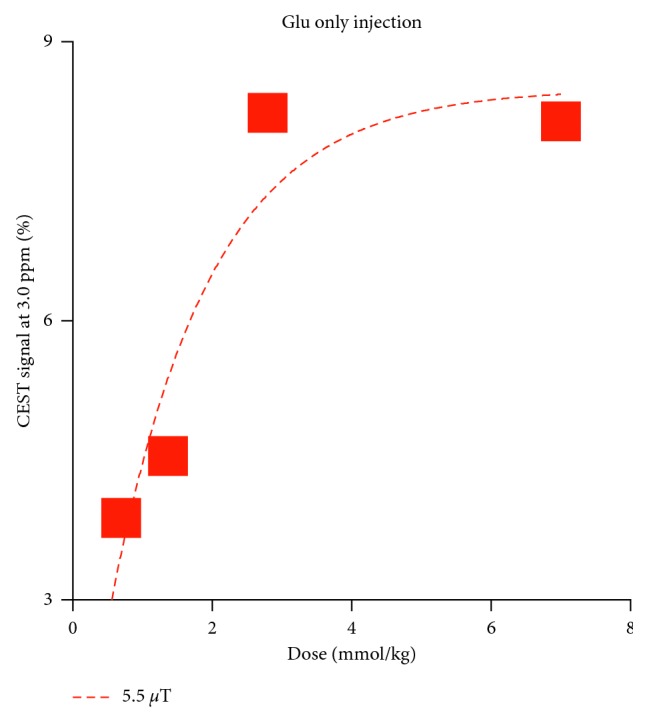
The CEST signals measured at 3.0 ppm peaked at around 10 min after injection of the Glu alone solution with different doses (0.7, 1.4, 2.8, and 7.0 mmol/kg). These maximum CEST signals in the renal medulla increased in a dose-dependent manner and plateaued after 2.8 mmol/kg.

**Figure 7:**
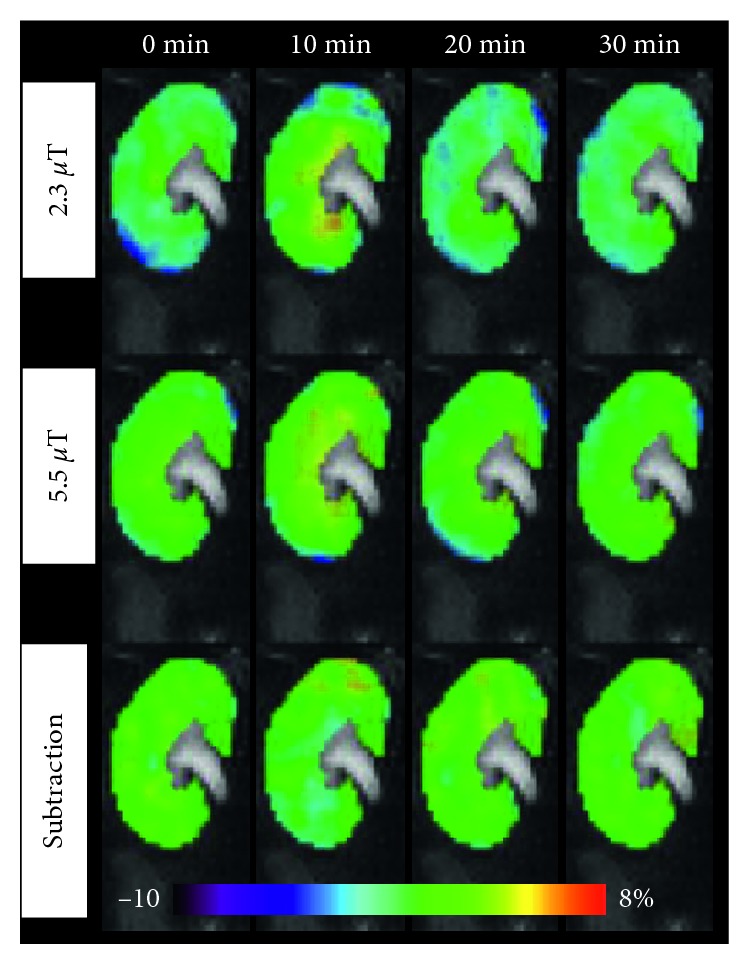
These are a set of representative coronal CEST maps at 3.0 ppm superimposed on T2-weighted images obtained with the presaturation amplitude of 2.3 *µ*T and 5.5 *µ*T, and the subtracted maps between the two power levels before and after injection of the Glu plus high-Nic solutions. The CEST maps are relatively homogeneous over most of the kidney, but small changes are evident after injection of mixed solution, especially in the medulla.

**Figure 8:**
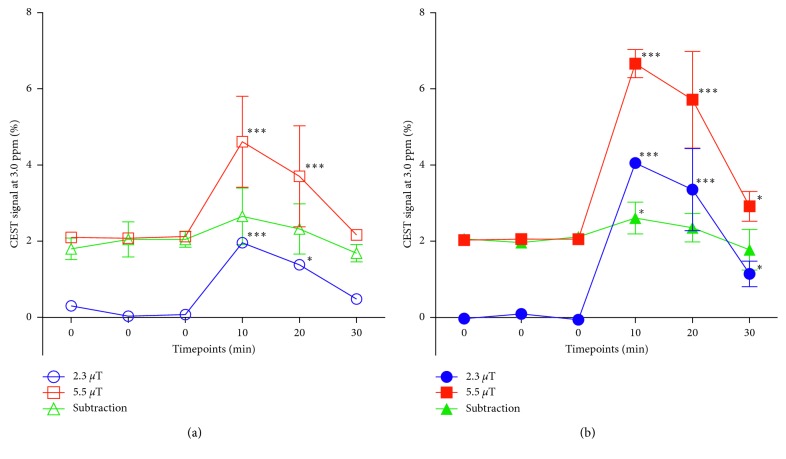
The temporal changes in the CEST signals at 3.0 ppm in the renal medulla are shown over 0 to 30 min after injection of Glu plus either low-Nic (a) and high-Nic (b) solutions. All CEST signals peaked at 10 min and decreased nearly back to baseline levels at 30 min after both injections. Although the injected dose of Glu was the same in both cases, the CEST signal was higher in the high-Nic group compared to that in the low-Nic group. By contrast, the subtracted CEST images showed similar levels of the CEST signals at all time points between the two injection groups (green lines). ^∗^*p* < 0.05 and ^∗∗∗^*p* < 0.001.

**Figure 9:**
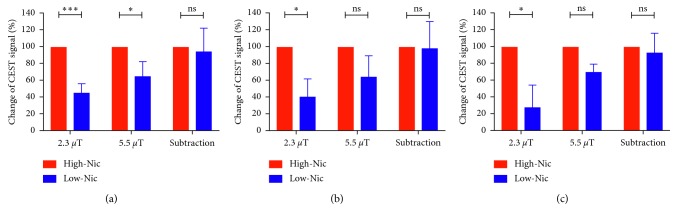
The CEST signals at 3.0 ppm in the low-Nic group showed substantially lower than those in the high-Nic group (ca. 40–60%) at 10 and 20 min with both presaturation powers. On the other hand, the CEST signals between the injection groups were almost same in the subtracted images. ^∗^*p* < 0.05 and ^∗∗∗^*p* < 0.001. (a) 10 min. (b) 20 min. (c) 30 min.

In the pilot study, to find an appropriate Glu dose, the CEST signal was measured at 3.0 ppm peaked at approximatly 10 min after injection of the Glu alone solution with different doses. These maximum CEST signals in the renal medulla increased in a dose-dependent manner and plateaued after 2.8 mmol/kg (Figure 6). Hence, we selected a dose of 1.4 mmol/kg of Glu for the subsequent study.  Figure 7 shows a set of representative coronal CEST maps at 3.0 ppm superimposed on T2-weighted proton images obtained with the presaturation amplitude of 5.5 *µ*T and 2.3 *µ*T and the subtracted maps between the two power levels before and after injection of the Glu plus high-Nic. The CEST maps were relatively homogeneous over most of the kidney, but small changes were evident in the medulla.  Figure 8 shows the temporal changes in the CEST signals at 3.0 ppm in the medulla over 0 to 30 min after injection of Glu plus either low-Nic (Figure 8(a)) or high-Nic (Figure 8(b)), respectively. All CEST signals peaked at 10 min and decreased nearly back to baseline levels at 30 min after both injections. Although the injected dose of Glu was the same in both cases, the CEST signal at 3.0 ppm was higher in the high-Nic group compared to that in the low-Nic group (Figure 8). This indicates that the interference from Nic is quite substantial. In contrast, the subtracted CEST images, which should reflect largely Glu, show similar CEST intensities at 3.0 ppm at all time points in both the high-Nic and low-Nic groups (Figure 8, green-colored lines). The Glu signals in the low-Nic group were substantially lower than those in the high-Nic group (ca. 40–60%) at 10 and 20 min with both presaturation powers (Figure 9). On the other hand, the Glu signals between the injection groups were almost same in the subtracted images. The results suggest that this subtraction method does reflect a true Glu signal without interference from Nic. Thus, the PPAP-CEST method increased the independence of the Glu signal making it less dependent upon other tissue metabolites.

## 4. Discussion

We first evaluated whether there was interference among the CEST signals of Nic, Glu, Cre, and Glc measured at 3.5, 3.0, 2.0, and 1.0 ppm, respectively, in phantoms. Not surprisingly, we observed that the CEST signals measured at each frequency were affected by a change in concentration of any one of the other exchanging molecular components. Based upon the presaturation pulse-dependent signal behavior, we developed a method to reduce such interferences on the GluCEST signal by the presence of three other exchanging species (Nic, Cre, and Glc) using two different presaturation amplitudes, 2.3 *µ*T and 5.5 *µ*T. Those results further confirmed that the PPAP-CEST method was able to increase the independence of the Glu signal in the animal study.

We selected the four substances that have been focused in several recent CEST studies [7, 9–11, 16–19]. Since it is not realistic to measure each CEST signal separately in tissues, we first measured *z*-spectra from the individual CEST signal in phantom that contained either low or high concentration of each substance. By summing four of these actually measured eight *z*-spectra (4 substances × 2 concentrations), we investigated the independence of each CEST signal in the five virtual conditions as follows: where all four substances mixed in high concentration (4-high) or where the concentration of any one of the four substances is reduced (Nic-low, Glu-low, Cre-low, and Glc-low, respectively) from the 4-high. The results revealed that all CEST signals were affected by a change in concentration of any one of the remaining exchanging species.

Regarding the evaluation of interference in the virtual mixtures, we could estimate the effect in any combinations of the substances, once we actually measured only 4 substances at 2 different concentrations. Using this method, we can save the substances and time for preparation of phantoms, data acquisition, and processing. Further, we can avoid any confusion in interpretation of the results due to possible errors in preparation of the substances among the phantoms. On the other hand, our virtual results could differ somewhat from the results in the phantoms containing those substances because molecular interactions between substances could alter the proton exchange rates. Hence, we additionally prepared a phantom that included all four substances in the same high concentrations (real 4-high) and compared its *z*-spectrum with the virtual 4-high *z*-spectrum. Since the MTR_asym_ from these two *z*-spectra were very similar, we believe that our results in this study are reasonable.

To extract a target CEST signal, several fitting methods have been suggested [10, 25]. A comparison of fitting methods showed a similar level of interference among the four exchanging components. The level of the interference may depend upon mathematical functions which would be applied for fitting, although it seems difficult to obviate the interference completely as the peaks of the substances on the *z*-spectra are broad and the spectral resolution of the *z*-spectra is usually low. This result indicates that one must take into account interferences among all possible CEST signals to properly interpret changes in the signal of any targeted exchanging species.

In the second phantom study, we examined the CEST signal of each component when the power of the presaturation pulse was changed by using different amplitudes and pulse durations. The CEST signals of Glu and Glc were more responsive to an increase in pulse amplitude compared to Nic and Cre. This reflects differences in proton exchange rates among these four species. The reported proton exchange rates for Glu (∼5,500 s^−1^, pH 7.0, 37°C, 7 T [9], and amino protons) and Glc (∼1,500 s^−1^, pH 7.3, 37°C, 11.7 T [11], and hydroxyl protons) are substantially faster compared to those of Nic (∼30 s^−1^, rat brain, 4.7 T [26], and amide protons) and Cre (∼950 s^−1^, pH 7.0, 37°C, 9.4 T [17], and guanidine protons). It is known that when proton exchange is sufficiently fast, more saturated spins can be transferred to bulk water as the amplitude increases (amplitude dependent). Conversely, when proton exchange is slower, the duration of presaturation becomes rate-limiting.

Glu is a major excitatory neurotransmitter in the brain, and endogenous Glu is circulating in plasma in normal condition [27] to play a central role for detoxifying ammonia in the kidney [28]. Hence, it would be clinically important to accurately quantify the Glu concentration ([Glu]) in tissues. For these reasons, we focused on the Glu signal to test whether the PPAP-CEST method might be able to increase independence of the Glu signal from other potentially interfering exchanging species. This was checked by collecting a pair of CEST images using two different pulse amplitudes, one at low power and the other at high power followed by subtraction of the two images. In the phantom study, we subtracted the result with 2.3 *µ*T presaturation pulse from the result with 5.5 *µ*T presaturation pulse and found that the amplitude-dependent Glu signal was enhanced. As demonstrated in the study, the method worked well in phantoms presumably because the Glu (3.0 ppm) has the highest exchange rate and the next neighboring Nic (3.5 ppm) and Cre (2.0 ppm) signals on both sides of frequency have much lower exchange rate [9, 29]. Glc (1.0 ppm) is another amplitude-dependent substance; however, the frequency of Glc is far enough from Glu (3.0 ppm). Indeed, the Glu signal was lowered by 0.5% when the concentration of Glc was reduced (Figure 3(b)). Therefore, we assume that the effect on the Glu signal is trivial if Glc would be injected together.

To detect the Glu *in vivo*, GluCEST was studied in a rat model of brain tumor by Cai et al. [9]. The authors reported that GluCEST detected an increased CEST signal in the tumor after intravenous injection of Glu. They confirmed that the increased signal was the Glu signal using magnetic resonance spectroscopy. Since exogenous Glu cannot pass the intact blood-brain barrier [30], we tested whether the same PPAP-CEST method worked *in vivo* to increase the independence of the Glu signal after ip injection of the Glu plus Nic solution in the renal medulla in mice. When we injected Glu plus either the low-Nic or high-Nic, the CEST signal increased immediately after ip injection of the mixed solutions and peaked at 10 min and gradually decreased up to 30 min in images collected at low and high pulse amplitude and in the subtracted images. One critical question is whether the signal changes we observed reflected “true concentration change of glutamate.” In the kidney, almost all Glu and Nic in the plasma/blood vessels pass across the glomerular capsule and go into the proximal and distal tubules where almost 80% of Glu/Nic is reabsorbed back into blood and the remaining ca. 20% is excreted into the urinary tracts in the kidney [27]. These Glu/Nic kinetics were evident in CEST spectra of the renal medulla in this study. Hence, it can be assumed that the concentration of these metabolites in the observed area largely reflects their concentration in plasma [27]. Regarding plasma concentration, it was reported that the residual plasma [Glu] is 0.122 mM under normal physiological conditions but can increase up to 36-fold after subcutaneous injection of Glu at a dose of 1.68 mmol/kg at 15 min and then gradually decrease over time [31]. In our study, we injected Glu ip at a dose of 1.4 mmol/kg; thus, it is reasonable to assume that the maximum [Glu] in plasma could be achieved to the same level (36-fold or somewhat less) somewhere around 15 min after injection. On the other hand, it was also reported that the residual concentration of Nic ([Nic]) in plasma was 0.0055 mM in mice and can be increased up to 128-fold after an ip injection of 0.8 mmol/kg and up to 840-fold after an ip injection of 4.0 mmol/kg [32]. In our study, we injected 0.7 mmol/kg in the low-Nic solution and 5.6 mmol/kg in the high-Nic solution, so the increase of Nic in plasma can be estimated to increase 112-fold and 1176-fold, respectively. Second, regarding Nic, the maximum % of Nic in urine of mice after ip injection of radioisotope of Nic (Nic-7-^14^C) was observed between 0 and 10 min, and it decreased over time [33]. Although this previous result was evaluated in urine, we assume that the temporal concentration change of Nic was similar as in plasma. For these reasons, the Glu signal we observed could be assumed to reflect the real temporal change of [Glu] and [Nic] in the renal medulla in mice.

With each power of the presaturation pulse, however, the CEST signals at 3.0 ppm at each time point after injection of the high-Nic were always higher than those after injection of the low-Nic, although the injection dose of Glu was same between the solutions. By contrast, the CEST signals at 3.0 ppm were the same at each time point (2.7% versus 2.6% at 10 min, 2.3% versus 2.4% at 20 min, and 1.7% versus 1.8% at 30 min, respectively) in the PPAP-CEST method, whether injected low-Nic or high-Nic. Furthermore, the CEST signal at 3.0 ppm was 2.9%, 2.4%, and 2.0% at each time point in the PPAP method after injection of Glu alone at a dose of 1.4 mmol/kg. Our results indicated that the PPAP-CEST method diminished the interference from the Nic signal and increased the independence of the Glu signal.

The exchange rates for these substances in phantom must be different from those in the tissue and may change depending upon the local tissue conditions (e.g., temperature, pH, and concentration). However, it is reasonable to assume that the order of the exchange rates for these metabolites would remain the same, with the amino acid the highest and the amide and guanidine much slower. We used 2.3 *µ*T and 5.5 *µ*T based on the phantom results, but this combination may not be optimal. The chosen power amplitudes or combinations of amplitudes (it may be better to use more than two powers) should be carefully optimized in each PPAP-CEST experiment for each target metabolite in different tissues to optimize the observed CEST signal *in vivo*. The PPAP-CEST method intrinsically reduces the sensitivity of the target signal because it does require subtraction of images. This means that the method is most effective when the CEST signal of the molecule of interest is large to begin with and PPAP-CEST is used to largely purify the signal from other interfering substances. Since the method also requires multiple presaturation pulses, fast imaging methods such as key-hole acquisition [34], compressed sensing [35], or 3D acquisitions [36] should be considered since the total imaging time for the PPAP-CEST method becomes longer as more presaturation pulses are used.

## 5. Conclusion

The PPAP-CEST method can be used to cleanse or remove contributions from other exchanging species in the same sample. This method was shown to improve the independence of the GluCEST signal *in vivo*. Although the optimal combination of the presaturation pulses were carefully considered, the PPAP-CEST method has the potential to increase the independence of any target CEST signal *in vivo* by choosing the appropriate combination of pulse amplitudes and pulse durations.
